# A successful approach to conrol burning mouth syndrome using matricaria recutita and cognitive therapy

**DOI:** 10.4317/jced.54686

**Published:** 2018-05-01

**Authors:** Alessandra-Maria-Ceolin Milani, Carmen-Lúcia-Rodrigues Macedo, Mariana-De-Carlo Bello, Celso-Afonso Klein-Júnior, Rubem-Beraldo dos Santos

**Affiliations:** 1 Student, School of Dentistry, Lutheran University of Brazil-Cachoeira do Sul; 2 DDS, MSc, Professor of the School of Dentistry, Lutheran University of Brazil-Cachoeira do Sul; 3 DDS, MSc, PhD Professor of the School of Dentistry, Lutheran University of Brazil-Cachoeira do Sul; 4 DDS, PhD Professor of the School of Dentistry, Oral Medicine Department , Lutheran University of Brazil-Cachoeira do Sul

## Abstract

The burning mouth syndrome (BMS) has no specific clinical and laboratory signs. Its etiology is yet to be elucidated, but it is considered to be affected by multifactorial, psychological, and local and systemic factors. This condition is considered of great morbidity, and the main complaint of patients maybe associated with xerostomia, thirst, and altered taste. The present study aims to report two cases of BMS and to evaluate the outcome of cognitive therapy (CT) plus phytotherapy in the control of BMS. The patients were female, Caucasian, and aged between 58 and 69 years. The most BMS-affected anatomical parts were the lips and the tongue. In the clinical approach, oral and systemic evaluation, and disease management with CT plus chamomile tea were done. The patients were reassured, and their response to therapy one year after was found to be excellent despite few exacerbations in periods of great emotional stress. Thus, we conclude that psychological treatment is vital in the management of BMS, as CT, along with Matricaria recutita phytotherapy, displayed excellent results in the control of BMS.

** Key words:**Anxiety, chamomile tea, xerostomia, psychosomatic.

## Introduction

The burning mouth syndrome (BMS) presents with symptoms of burning or pain in the oral cavity, especially in the tongue, lips, and hard and soft palates, without clinical sign of systemic or oral disease (1). The BMS prevalence can range from 0.7% to 18% according to different studies (2). Women with ages ranging from 54 to 71 years is the most common age group affected by BMS; therefore, it is less frequently seen in males and 25 to 97 years old age group (3,4).

The etiology of BMS is not yet clear, but it is related to local, psychological, and systemic factors (5-7). Among the local factors listed are maladaptation of prosthesis, parafunctional oral habits, oral candidiasis, and damage to periphery nerves (8). Changes in estrogen production lead to different reaction of the receptors in the oral mucosa, which result in burning sensation. In addition, iron deficiency anemia and deficiency of complex B vitamins and folic acid, could be related to BMS (1).

Normally, the treatment of BMS is as complex as its etiology. In this context, several therapies, either topical or systemic, such as cited laser therapy, acupuncture, clonazepam, capsaicin, and amitriptyline, have been attempted to manage BMS (9,10).

None of above options is considered as a definitive solution; hence, other possibilities are searched. In line with this, cognitive therapy (CT) is used to help the patient understand the nature of their symptoms and help him realize that the burning sensation can be a mechanism to help him cope with emotional problems (5,6,11,12). On the other hand, chamomile (Matricaria recutita) could be useful in oral diseases due to its antioxidant, antimicrobial, and anti-inflammatory properties. Furthermore, its use in controlling BMS has been previously reported (13-15).

Considering the bibliographical background, the present study aims to report the management of two BMS cases through the combination of CT and chamomile tea to control BMS.

## Case Reports

Case 1: A 68-year-old, Caucasian woman complaining of dryness and burning sensation in lower lip and right side of tongue was examined in the Oral Medicine Department of Lutheran University of Brazil. She was previously diagnosed with anxiety and is currently under psychiatric care. Oral examination and laboratory results, such as full blood count, serum glucose, folic acid, vitamin B12, folate, and iron levels in the serum, were unremarkable. The patient recorded a score of 8 over 10 on the visual analogue scale (VAS) (10,11,14). The symptoms she was experiencing, and its emotional impact, were explained to her. Furthermore, she was reassured that she does not have oral cancer. She was then diagnosed with Type I or idiopathic BMS (9). For topical therapy, she was recommended to drink large volume of water and chamomile tea without sugar. The tea must be kept in the mouth for three minutes, four times a day. A letter explaining BMS and the possible use of CT for treatment purposes was sent to her physician, and he agreed with the proposed therapy. After 14 days, she returned to the clinic for re-examination wherein she reported a significant reduction in burning sensation and an improved general well-being. She scored 4 over 10 on VAS. The therapy was continued for one year, with visits for 15 days for the first two months and monthly after. In the last visit, she recorded 3 over 10 on VAS. In two months, she recorded zero and one.

Case 2: A 59-year-old Caucasian female consulted for help in the same department due to burning sensation on the lips that she has been experiencing for a year and a half. She also reported that in the last three months, the burning sensation started in the morning and lasted all day. She has a history of controlled hypertension that is managed with proper diet, exercise, and 50-mg/day of Losartan Potassium, which did not result in hyposalivation (0.52-mL/ min no stimulated salivary flow rate). She did not report with xerostomia as well. The oral and laboratory examinations were unremarkable and showed only variations in the normality of oral mucosa, tongue varicosities, and Fordyce’s granules. She was diagnosed with Type I or idiopathic BMS (9). She recorded a score of 9 over 10 on VAS. She was advised to use topical chamomile, as previously mentioned, and was referred to psychological treatment, in which the psychologist employed CT. The patient returned to the clinic for re-examination after 14 days and reported significant reduction in burning sensation and improved well-being. Moreover, her score on VAS was decreased to three. The schedule of re-examination was same as that of patient 1. In the last visit, the patient reported 2 over 10 on the VAS, and in a month, the reported score was zero. However, four months, she reported 1 over 10 on VAS, as the burning sensation increased during very few periods of anxiety.

## Discussion

In the diagnosis of BMS, the oral mucosa does not present with alterations that could justify the symptoms but only variations in normality such as Fordyce´s Granules or tongue varicosities, as seen in-patient 2. In this context, the examiner must be prepared to disregard other oral diseases (1,3). In both cases, no irritability and mouth dryness, as usually seen in BMS, were reported (10).

In accordance with literature, the patients were women in menopausal period. The same was observed when the women reported that their most frequently affected anatomical sites were the lips and tongue (6-8).

Although many questions about the etiology of BMS have not yet been answered, the psychological nature of this disease can be found in literature (11,12); in fact, in the present study, the patients had fear of cancer, anxiety, and high level of self-reported stress. In both cases, CT contributed in increasing their well-being, and they showed confidence in the approach proposed by Oral Medicine Department.

Numerous modalities, such as, medicines, laser therapy, acupuncture and, of course, psychological treatment, can be employed in the management of BMS (9). In this study, a novel approach in BMS is the combination of CT and chamomile tea. The use of M. recutita infusion was recommended for keeping the mouth wet. Furthermore, it is cost-efficient and has no reported adverse effects; and its usage is in accordance with the Brazilian culture, thus excellent patient compliance is expected. In addition, other advantages of this chamomile include dental plaque control, oral aphthae and gingivitis treatment (13,15). With regards to its specific use in BMS, Valenzuela *et al.* performed a prospective, randomized, placebo-controlled, double-blind study that included 62 patients with idiopathic BMS (14). No significant difference was observed in VAS scores between the placebo group and the chamomile-treated group; We believe that the probable difference between the said study and the present report is that Valenzuela *et al.* used a 2% chamomile gel once a day, whereas we used chamomile infusion four times daily with CT.

As for the use of CT for BMS control, in one classical study, the authors compared 15 patients under CT with 15 patients under placebo regimen as all of them suffered from resistant BMS (11). Similarly to the results of the present study, the CT-treated group was more benefited with CT therapy, as in a 6-month follow-up, the VAS score of the CT-treated group was lower as compared with that of the placebo group. This findings were confirmed by others study and scientifically supported the present method (10,12).

The diagnosis, classification, and management of BMS are complex, thus, a multidisciplinary approach as used in the present study, individuality of therapies, good physician-patient relationship, active understanding of patient about the nature of their disease and fight against fear of cancer appear to be very useful. For the nearly future, if the method presented in this case report gets published, it will be used as a validated scientific reference in a clinical trial to compare a group of chamomile plus CT therapy with a placebo group in order to bring more light over the ideal BMS treatment. It is necessary to say that part of this method is already being used in Oral Medicine Service in Southern Brazil more than a decade.

By taking into consideration the results of reported cases, we conclude that CT, along with M. recutita infusion, showed promising results in the management of BMS.
